# Event-Related Brain Potentials Associated With the Olfactory-Visual Stroop Effect and Its Modulation by Olfactory-Induced Emotional States

**DOI:** 10.3389/fpsyg.2020.00583

**Published:** 2020-04-09

**Authors:** Miaomiao Xu, Nicolas Dupuis-Roy, Jun Jiang, Chengyao Guo, Xiao Xiao

**Affiliations:** ^1^School of Public Health and Management, Chongqing Medical University, Chongqing, China; ^2^Research Center for Medicine and Social Development, Chongqing Medical University, Chongqing, China; ^3^Innovation Center for Social Risk Governance in Health, Chongqing Medical University, Chongqing, China; ^4^Départment de Psychologie, Université de Montréal, Montreal, QC, Canada; ^5^Department of Basic Psychology, School of Psychology, Third Military Medical University, Chongqing, China

**Keywords:** olfactory-visual cross-modality, Stroop effect, event-related brain potentials, sensory-induced emotional states, ND350-550

## Abstract

This study investigated the event-related brain potentials associated with the olfactory-visual cross-modal Stroop effect and its modulation by olfactory-induced and self-reported affective states. Eighteen healthy participants were presented with an olfactory stimulus and the image of a plant, and they had to categorize the olfactory attribute of the image as “aromatic” or “pungent” by pressing the relevant button as quickly as possible. The type of olfactory-visual stimuli (congruent or incongruent) and the valence of the olfactory-induced emotional states (positive or negative) were manipulated following a 2 × 2 factorial design. Interference effects were observed at the behavioral and the electrophysiological levels: response times recorded in the incongruent condition were higher than those observed in the congruent condition; an incongruent minus congruent negative difference component was discovered between 350 and 550 ms after stimulus onset in the negative—but not in the positive—olfactory-induced emotional state condition. This ND350-550 component was interpreted as reflecting the amount of selective attention involved in the olfactory-visual cross-modal Stroop effect. These results are also consistent with a facilitatory effect of positive emotional state on selective attention which could reduce brain potentials associated with the cross-modal interference effect.

## Introduction

Selective attention involves filtering out irrelevant information from the surrounding environment and focusing on the task at hand. Many situations in everyday life require selective attention. For example, bus drivers must ignore distracting neon signs on surrounding buildings and focus on traffic lights in order to take appropriate decisions at an intersection. Similarly, a sprinter at the Olympic Games must filter out the noise from the crowd in order to perceive and react promptly to the starter gun. Selective attention also plays a central role in animals. For instance, sniffer dogs used in the aftermath of an earthquake need to ignore multiple odors coming out of the ruins in order to detect survivors. Selective attention is the foundation of adaptive behaviors.

One experimental paradigm that has been extensively used to study selective attention is the Stroop task. In its original form, the Stroop task consists in naming, as fast as possible, the color in which a word is printed, ignoring the word itself. When the word designates a color that does not match the ink color in which it is printed, we expect the average response time to be longer than the average response time recorded in a control condition such as naming the color of a solid color square (Stroop, 1935).

Nowadays, studies using the “classic Stroop task” usually refer to the following paradigm: a color word (e.g., ‘blue,’ ‘red,’ ‘green,’ etc.) is displayed on a screen in a congruent (e.g., the word ‘red’ in red font) or in an incongruent font color (e.g., the word ‘red’ in green font). Subjects are required to name, as quickly as possible, the font color of the color word. The Stroop effect refers to the longer response latency observed in incongruent than in congruent conditions.

Since John Ridley Stroop (1935) reported his initial experiment, many variations of this paradigms have been proposed, including the day–night Stroop (Ikeda et al., 2014), the emotional Stroop (Fehr et al., 2006) and the numerical Stroop (Pagano and Mazza, 2013). These different variations have been used to investigate the many facets of selective attention including the nature of its underlying processes, its neural basis, its development, and its disorders. Many researchers have used the event-related potentials (ERPs) technique to examine the neural basis and the time course of selective attention in a Stroop task. Most of these studies have found a significant negative incongruent-vs.-congruent difference wave between 350 and 550 ms post-stimulus (Liotti et al., 2000; Markela-Lerenc et al., 2004; Qiu et al., 2006).

In daily life, selective attention often takes place through different sensory channels. For example, to be able to read the newspaper in a cafeteria, people need to ignore conversations at nearby tables. Empirical studies have also revealed Stroop-like effects occurring between sensory modalities. Elliott et al. (1998) reported that the participants named a color patch faster when it was presented with a congruent auditory color word. Pauli et al. (1999) observed a cross-modality priming effect between olfaction and vision: they showed that the presence of a pleasant or unpleasant odor interfered with the performance on a color-naming Stroop task that included odor-congruent and odor-incongruent words. Similarly, White and Prescott (2007) found that the taste of a gustatory stimulus was more quickly identified when it was presented with a congruent odor (e.g., sweet taste and strawberry odor) than with an incongruent one (e.g., sour taste and strawberry odor). Relatedly, Xiao et al. (2014) observed that participants identified an image more quickly when it was shown with a congruent gustatory stimulus (e.g., sweet taste and image of cake) than with an incongruent one (e.g., sweet taste and image of lemon). Also, their ERP analyses revealed this cross-modal Stroop effect was associated with an incongruent-vs.-congruent negative difference (ND) wave occurring between 430 and 620 ms post-stimulus. Although several studies examined cross-modal Stroop effects in humans, none has specifically explored, to the best of our knowledge, ERPs associated with the olfactory-visual Stroop effect.

Emotional states are affective states which usually do not last long. Such transitory affective states can nonetheless impact on our everyday activities via their interactions with our cognitive processes (Mitchell and Phillips, 2007). For example, a students’ attention level can decrease suddenly after receiving the unexpected news of a failed test or a perfect score. In fact, many empirical studies have demonstrated how a positive emotional state can reduce performance during a unimodal Stroop task by decreasing focused attention (Phillips et al., 2002; Rowe et al., 2007).

According to previous studies (Delplanque et al., 2017; Seubert et al., 2017), olfactory stimuli could induce a positive (or negative) emotional state. Colognes could induce a positive emotional state (Schiffman et al., 1995), whereas hydrogen sulfide might induce an unpleasant one (Finkelmeyer et al., 2010). In addition, citrus coniferous scents usually make people feel tense and hostile, whereas floral woody scents usually make people feel relaxed (Retiveau et al., 2004). This also seems consistent with widespread meditation practices in which people burn incense sticks presumably to induce a pleasant and relaxed affective state. This could in turn help them keep their focus.

In most electrophysiological studies mentioned above, stimuli of positive and negative valences were intermingled in the same condition, which may have concealed the impact of sensory-induced emotional states on the cross-modal Stroop effect. Many studies have demonstrated the impacts of sensory-induced emotional states on the unimodal Stroop effect (Phillips et al., 2002; Rowe et al., 2007; Yuan et al., 2011). However, the impacts of sensory-induced emotional states on the cross-modal Stroop effect remain largely unknown. The aim of the current study was thus twofold: to discover ERPs associated with the olfactory-visual Stroop effect; and to examine the impact of olfactory-induced emotional states on the olfactory-visual cross-modal Stroop effect.

Most researchers agree that the unimodal Stroop effect involves an early perceptual/attentional stage and a later cognitive control stage (Qiu et al., 2006; Yuan et al., 2011). Each of these stages is indexed by a different ERP: the early perceptual/attentional stage was shown to be associated to a P2 component (Yuan et al., 2011), while the latter cognitive control stage—as explained above—was found to be associated to an incongruent-minus-congruent ND waveform occurring between 350 and 550 ms (Liotti et al., 2000; Markela-Lerenc et al., 2004; Qiu et al., 2006). Given that the neural mechanisms involved in multisensory information processing has already been shown to be different from those elicited by inputs from a single sensory modality (Thesen et al., 2004; Macaluso and Driver, 2005; Macaluso, 2006), we generally expected our olfactory-visual Stroop task to impact on both the early P2 and latter ND waveform.

The incongruent-vs.-congruent ND waveform recorded in unimodal Stroop tasks usually occurs at slightly lower latency than in cross-modal Stroop tasks. For instance, Xiao et al. (2014) observed a negative incongruent-vs.-congruent ND waveform between 430 and 620 ms post-stimulus in a cross-modal taste-visual Stroop task. In the classic unimodal Stroop task, the visual stimuli (e.g., the print color and the word itself) that produce the effect are usually displayed at the same location on the visual field and processed by a single primary cortex. By contrast, the stimuli in the cross-modal Stroop task are presented at different physical locations on different sensory channels (e.g., taste and vision) and processed through different primary cortices. Therefore, it has been proposed that interference effect indexed by the incongruent-vs.-congruent ND waveform in cross-modal Stroop tasks could be delayed as a result of the integration of cross-modal sensory signals (Wang et al., 2011; Xiao et al., 2014). Consistent with this view, we also expected a delayed ND waveform in our cross-modal olfactory-visual Stroop task. Concerning the P2, we did not have detailed prior expectations and thus regarded this matter as an empirical one.

## Materials and Methods

### Participants

Seventy university students aged between 20 and 25 years (35 males 35 females; *M* = 22.6, *SD* = 1.6) were recruited in the city of Chongqing (China). They needed to evaluate their emotional states after being submitted to each of the six visual stimuli and the two olfactory stimuli. More specifically, they first had to indicate, on a seven-point Likert scale going from −3 (highly unpleasant) to 3 (highly pleasant), how they felt about each one of six images of plants: jasmine, narcissus, plum blossom, scallion, garlic, and shallot. Sixty-eight of them rated all the images as neutral. These 68 participants then had to indicate, using the same scale, how they felt about each olfactory stimulus: 5 ml of GF brand cologne (components: ethanol, water, flavor and butadiene toluene; concentration: flavor accounts for 3% of entire volume; Fragrance: Floral) and 15 ml of an onion-garlic mixture (which was made by mixing 500 g of chopped onion with 500 g of chopped garlic and 100 ml of water using a blender.). Eighteen participants rated the perfume as highly pleasant and the onion-garlic mixture as highly unpleasant. Only these 18 participants (9 men and 9 women) were kept for the rest of the study. Note that these participants rated the olfactory and visual stimuli 1 week before and right before the Stroop task. The ratings did not change.

The selected volunteers were aged between 21 and 25 years (*M* = 22.7; *SD* = 1.3). All of them were healthy, right-handed, and all had normal or corrected-to-normal vision. None of them reported any allergy to perfume, onion or garlic. None of them reported suffering from colds, sinus problems or asthma. Following a short olfactory test in which participants were asked to describe the smell coming out of identical and opaque vials, none of them showed or reported any symptoms related to hyposmia or anosmia. The experiment was approved by the Ethics Committee of Chongqing Medical University. A written informed consent was obtained from all participants prior to the experiment, and a monetary compensation was given to them after its completion. All volunteers were required to clean their nose with a saline solution before the experiment.

### Stimuli

The visual stimuli included six digital images: three images of aromatic flowers (jasmine flower, narcissus flower, and plum flower) and three images of pungent plants (scallion, garlic and shallot). For validation purposes, an extra 70 student (36 males and 34 females) were recruited and asked to categorize the olfactory attribute of the images as “aromatic” or “pungent.” None of them made a mistake. Before the beginning of the experiment, the selected group of 18 participants underwent a familiarization session with the images in which each participant saw each image twice. Then, they had to categorize the odor associated with the plants in the image as “aromatic” or “pungent.” No error was made during this categorization task. To evoke the aromatic or pungent smells, 5 ml of perfume or 15 ml of the onion-garlic mixture was put in a Bunsen beaker, which was then positioned 8 cm below the nose of the participant. These volumes were chosen based on a small pilot study in which 10 subjects, who did not participate in the final study, were asked to rate the intensity level of and their familiarity with each olfactory stimulus. Final results indicated that there were no noticeable differences between the two chosen olfactory stimuli in terms of intensity and familiarity.

### Procedure

Participants were seated in a quiet room at a distance of 60 cm from the computer monitor and were required to wear protective glasses to prevent accidental contacts with the olfactory solutions. They were instructed to avoid blinking or moving their eyes or mouth and to keep their eyes fixated on the monitor, rather than looking down at their fingers, during the task. Prior to the beginning of the experiment, participants were trained to breathe through their nose without concomitant oral movements and the deglutition. Just before the beginning of a block of trials, the experimenter put either 5 ml of perfume or 15 ml of the onion-garlic mixture below the participant’s nose and instructed him/her to keep his/her head still for the whole duration of the block. To facilitate the diffusion of the odorous solution from the beaker, two electric fans located on each side of the participant were turned on.

Each block comprised 60 trials. A single olfactory stimulus, either aromatic or pungent, was presented on each block to induce a consistent positive or negative emotional state. In contrast, both types of images (i.e., of aromatic and pungent plants/flowers) were shown on every block and were assumed to have no impact on the emotional states of participants. Before the beginning of each block, a given olfactory solution was placed under the participant’s nose and the two electric fans were turned on. The participant then had to wait for 30 s before the beginning of the first trial so that a sufficient amount of olfactory solution could diffuse into the air. Each trial began with a fixation cross (‘ + ’) that was displayed for 300 to 800 ms at the center of the screen. This duration was randomized across trials. Then, the image of a given plant was shown until the participant pressed a response key and for a maximum duration of 3000 ms. Participants were instructed to indicate, as quickly and accurately as possible, the odor of the plant depicted in the image by pressing the appropriate keyboard key. Images were shown in a random order. The olfactory stimulus was changed on each successive block to avoid desensitization. Between each block, participants had to take a 5-min break during which the air conditioner was turned on to get rid of the smell in the room and to avoid contamination between olfactory stimuli.

The order of olfactory stimuli was balanced: nine participants (5 men and 4 women) started with the aromatic smell block, whereas the rest started with the pungent smell block. For all selected participants, the pungent smell was associated with highly unpleasant feelings; the aromatic smell with highly pleasant feelings; and the images with neutral feelings. The valence of the olfactory-induced emotional states (negative/positive) and the congruency of the cross-modal stimuli (congruent/incongruent) were manipulated orthogonally to produce four experimental conditions: negative congruent (NC; pungent smell and image of pungent plant), negative incongruent (NI; pungent smell and image of aromatic plant), positive congruent (PC; aromatic smell and image of aromatic plant), positive incongruent (PI; aromatic smell and image of pungent plant). Participants had to complete four blocks of 60 trials each, that is 60 trials in each of the four experimental conditions.

### Electrophysiological Recording and Analysis

Brain electrical activity was measured from 64 sites on the scalp using tin electrodes mounted in an elastic cap (Brain Product, Brain Products GmbH, Stockdorfer, Gilching, Germany). The reference electrodes were on the left and right mastoids. The vertical electrooculogram (VEOG) was recorded with electrodes placed above and below the right eye, and the horizontal electrooculogram (HEOG) with electrodes placed on the outer canthi of each eye. All interelectrode impedance was maintained below 10 kΩ. The electroencephalogram (EEG) and electrooculogram (EOG) were amplified using a 0.05–100 Hz band-pass and continuously sampled at 500 Hz/channel for off-line analysis. Eye movement artifacts (blinks and eye movements) were rejected offline by using the Gratton et al. (1983) algorithm (Brain Vision Analyzer, Version, 1.05, Software, Brain Product GmbH). This algorithm corrects ocular artifacts by subtracting the voltages of the eye channels, multiplied by a channel-dependent correction factor, from the respective EEG channels. Trials with EOG artifacts (mean EOG voltage exceeding ± 80 μV) and those contaminated with artifacts due to amplifier clipping, bursts of electromyographic activity, or peak-to-peak deflection exceeding ± 80 μV were excluded from averaging. An automatic artifact rejection algorithm was used to detect artifact-contaminated trials.

**FIGURE 1:**
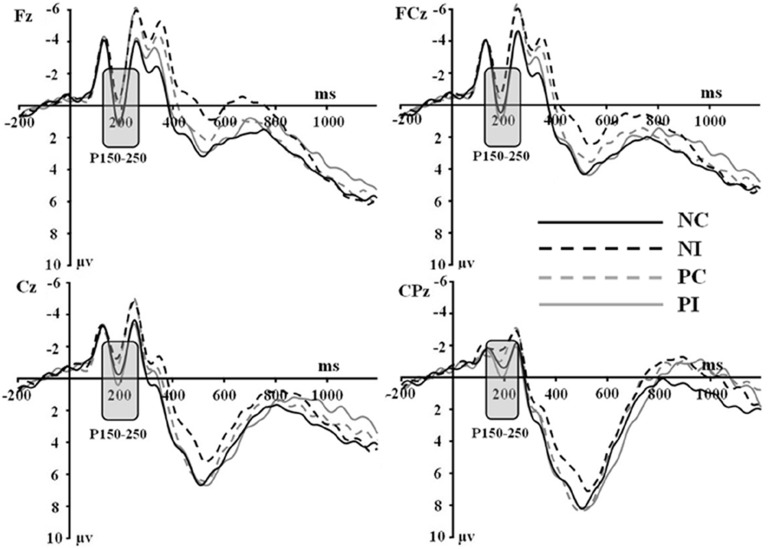
Grand average event-related potentials to NI condition, NC condition, PI condition, and PC condition at Fz, FCz, Cz, and CPz.

**FIGURE 2:**
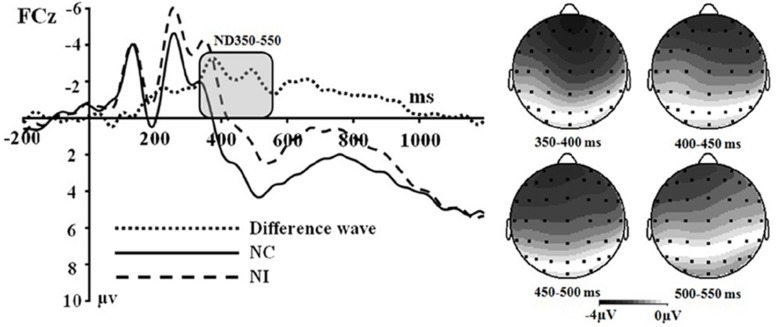
**(Left)** Grand average event-related potentials to NI condition, NC condition and the difference wave (NI – NC) at FCz. **(Right)** Topographical maps of the voltage amplitudes for NI condition versus NC condition difference wave in the time range 350 to 550 ms.

The averaged epoch for ERP lasted 1200 ms, going from 200 ms before visual stimulus onset to 1000 ms after visual stimulus onset. Segments with correct responses were averaged. At least 40 trials were available in each condition. Based on the ERPs grand averaged waveforms and topographical map (see Figures 1, 2), the following 25 electrodes were chosen for statistical analysis (Frontal: Fz, F1, F2, F3, and F4; Central: FCz, FC1, FC2, FC3, FC4, Cz, C1, C2, C3, C4, CPz, CP1, CP2, CP3, and CP4; Parietal: Pz, P1, P2, P3, and P4). For all analyses, the *p*-value was corrected for deviations according to Greenhouse–Geisser.

## Results

### Behavioral Results

Mean log-transformed RTs and percentages of accuracy for all conditions are summarized in Table 1. Repeated measures ANOVAs were performed on log-transformed RTs of accurate responses and accuracy in order to compare the effect of the olfactory-visual stimuli conditions (congruent vs. incongruent) and the olfactory-induced emotional states (positive vs. negative). No significant main effect or interaction was found in the ANOVA on accuracy, whereas a significant main effect of olfactory-visual stimuli condition was found in the ANOVA on log-transformed RTs [*F*(1,17) = 24.2, *p* = 0.00013] suggesting the participants spent a longer time processing stimuli in the incongruent condition than in the congruent condition. No interaction was found in the ANOVA on log-transformed RTs between the olfactory-visual stimuli condition and the olfactory-induced emotional states condition [*F*(1,17) = 0.12, *p* = 0.732].

**TABLE 1:** Descriptive statistics for behavioral data.

Condition	Log-transformed RT (ms)	Accuracy (%)
NI	2.86 ± 0.048	98.9 ± 1.7
NC	2.84 ± 0.051	98.5 ± 1.7
PI	2.86 ± 0.049	98.6 ± 1.8
PC	2.83 ± 0.045	98.6 ± 2.2

### Electrophysiological Scalp Data

Based on the ERPs grand-averaged waveforms (see Figures 1, 2 and Table 2) and previous studies, mean amplitudes calculated within two selected time windows—the 150–250 and 350–550 ms time windows—were analyzed using repeated-measures ANOVAs. The factors included in the analyses were two olfactory-visual stimuli conditions (incongruent vs. congruent), 25 electrode sites (Frontal: Fz, F1, F2, F3, and F4; Central: FCz, FC1, FC2, FC3, FC4, Cz, C1, C2, C3, C4, CPz, CP1, CP2, CP3, and CP4; Parietal: Pz, P1, P2, P3, and P4) and two olfactory-induced emotional states (positive and negative).

**TABLE 2:** Descriptive statistics for ERP data.

Condition	Mean amplitudes and SD of the ERPs between 150 and 250 ms (μV)	Mean amplitudes and SD of the ERPs between 350 and 550 ms (μV)
NI	−0.9 ± 1.74	2.26 ± 2.63
NC	−0.23 ± 1.91	3.58 ± 2.86
PI	0.1 ± 1.98	3.63 ± 2.45
PC	−0.69 ± 1.91	3.4 ± 2.55

First of all, there was no significant main effect of the electrodes, olfactory-visual stimuli condition and the olfactory-induced emotional reactions, whereas there was a significant two-way interaction between the olfactory-visual stimuli condition and the olfactory-induced emotional reactions within the 150–250 ms time window [*F*(1,17) = 11.23, *p* = 0.004]. The analysis of simple effects within the 150–250 ms latency window revealed a more positive deflection in the NC condition than in the NI condition [*F*(1,17) = 12.18, *p* = 0.003] and a more positive deflection in the PI condition than in the PC condition [*F*(1,17) = 6.78, *p* = 0.019]. No significant interaction was found between the electrode site and the other two factors (i.e., the olfactory-visual stimuli condition and the olfactory-induced emotional state conditions) within the 150–250 ms latency window.

Secondly, a two-way significant interaction was found between the olfactory-visual stimuli condition and the olfactory-induced emotional state conditions within the 350–550 ms time window [*F*(1,17) = 10.4, *p* = 0.005]. The simple effect analysis indicated that the incongruent condition elicited a significantly smaller positive ERP deflection than did the congruent condition in the negative olfactory-induced emotional state [*F*(1,17) = 12.22, *p* = 0.03], whereas no difference was observed in the positive olfactory-induced emotional state condition [*F*(1,17) = 1.61, *p* = 0.222]. Mean amplitudes were relatively more negative for NI than for NC condition. There was no significant main effect of the electrodes, the olfactory-visual stimuli condition and the olfactory-induced emotional reactions within the 350–550 ms time window.

## Discussion

This study aimed at better understanding how the selective attention required in an olfactory-visual Stroop task could be affected, at both the behavioral and neurological levels, by positive or negative self-reported emotional states induced by a pleasant or unpleasant odor. To achieve this goal, we recorded event-related brain potentials of 18 healthy participants during an olfactory-visual cross-modal Stroop task, and we manipulated the congruency of the olfactory-visual stimuli (congruent or incongruent) along with the valence of the olfactory-induced and self-reported emotional state (positive or negative). As expected, the cross-modal Stroop task produced interference effects at the behavioral and the electrophysiological levels. We observed higher response times in the incongruent condition than in the congruent condition. We also discovered an incongruent minus congruent negative difference component occurring between 350 and 550 ms after stimulus onset only in the negative—but not in the positive—olfactory-induced emotional state condition. Moreover, the P150-250 component found in the negative congruent condition was more positive than in the negative incongruent condition; and was more positive in the positive incongruent condition than in the positive congruent condition. Next, we discuss the implications of these findings.

Multiple ERP studies using unimodal and cross-modal Stroop tasks are consistent with our claim that the ND350-550 waveform observed in our olfactory-visual Stroop task reflects selective attention during incongruent cross-modal information processing. First, the incongruent-vs.-congruent difference ERP component recorded in classic unimodal Stroop tasks usually shows a negative deflection with a fronto-central topography that peaks between 350 and 550 ms post-stimulus, and this component has been associated with selective attention and cognitive control (Liotti et al., 2000; Markela-Lerenc et al., 2004; Qiu et al., 2006). Second, a similar ERP component has been reported in cross-modal Stroop tasks and has been interpreted as reflecting cross-modal cognitive control exerted through attentional processes (Wang et al., 2011; Xiao et al., 2014).

The ND waveforms reported here occurred slightly earlier—between 350 and 550 ms—than the ones reported in the taste-visual Stroop task (Xiao et al., 2014). Olfaction, perhaps the oldest sensory modality—phylogenetically speaking (Hosek and Freeman, 2001)—is unique among sensory modalities in its anatomical organization (Freeman, 2007). Unlike other sensory channels (e.g., vision, audition, or touch), bottom-up afferences from the olfactory receptors bypass the thalamic “first-order” relay neurons and directly influence a region of the olfactory (piriform) cortex (Gottfried and Zald, 2005). The speed of olfactory information far exceeds that of taste (Szyszka et al., 2012; Wallroth et al., 2018). Thus, one simple explanation for this earlier peak observed in the current study could be that the speed of olfactory information processing is faster than that of taste.

Contrarily to our expectations, the latency of the ND waveform recorded here is comparable to the latency of ND waves observed in unimodal Stroop tasks. However, we believe this could be partly explained by differences in the experimental procedure: the classic unimodal Stroop task usually involves four response options (i.e., red, yellow, green, and blue) whereas our cross-modal Stroop task only included two response options (i.e., aromatic and pungent). This difference in task difficulty might reduce (or even cancel) the expected latency difference due to cross-modality.

Another goal of the current study was to examine the impact of the valence of the olfactory-induced emotional states on the cross-modal Stroop effect. We approached this issue as an open empirical question and found a modulatory effect of sensory-induced emotional states on the olfactory-visual cross-modal Stroop effect. More specifically, the ND350-550 components associated with the olfactory-visual cross-modal Stroop effect were found in the negative olfactory-induced emotional states but not in the positive olfactory-induced emotional states. This result is consistent with some of our recent findings.

Xiao et al. (2018) used the fMRI technique to investigate brain activations related to conflict control in appetitive and aversive gustatory contexts during a taste-visual cross-modal pairing task. More specifically, participants were submitted to gustatory (e.g., sour or sweet taste) and visual (e.g., image of a lemon or ice cream) stimuli, and they had to decide, as quickly as possible, whether these stimuli matched (e.g., sour taste and image of lemon) or not (e.g., sour taste and image of ice cream). Blood oxygenation level-dependent (BOLD) contrasts between the mismatched and the matched conditions revealed an increased activity in the middle frontal gyrus. Significant activations were observed in the negative gustatory conditions but not in the positive gustatory conditions which suggested that the positive emotional states induced by the appetitive gustatory stimulation increased cognitive flexibility and improved cognitive control abilities. Given the similarities between the pairing task and the Stroop task—some researchers even consider them as the same paradigm (Wang et al., 2011)—we believe this hypothesis also applies to the current results: a positive sensory-induced emotional states has a facilitatory effect on cognitive control abilities and this facilitation translates into a reduction of the brain activity associated with the cross-modal information processing.

Notwithstanding, our results are also at odds with previous studies supporting the hypothesis that a positive emotional state impairs performance during the unimodal Stroop tasks (Phillips et al., 2002; Rowe et al., 2007). Especially, Yuan et al. (2011) examined the effect of the emotional states on ERPs associated with selective attention and cognitive control during a unimodal Stroop task. On each trial, the participants first listened to an audio excerpt that induced either a pleasant, neutral or unpleasant emotional state, and then performed a unimodal Stroop task. A single emotional state was assigned to each block of trials. Their behavioral results revealed that color-naming responses were faster during pleasant blocks than unpleasant (or neutral) blocks, but they did not find any significant interaction between the emotional states condition and the stimuli type (incongruent or congruent). Although they failed to demonstrate any facilitatory effect at the behavioral level, they did find a significant interaction on ERPs: the incongruent minus congruent ND waveform recorded within the 450–550 ms interval was more negative in the pleasant than in the unpleasant emotional state condition.

Although our results are at odds with these findings, some differences were expected given the evidence suggesting that different neural mechanisms are recruited during the unimodal and cross-modal Stroop tasks. The prefrontal cortex has been involved in both the unimodal (Liotti et al., 2000; Markela-Lerenc et al., 2004; Qiu et al., 2006) and the cross-modal Stroop effect (Xiao et al., 2014). In contrast, the parahippocampal gyrus has only been associated with the processing of discordant information in the cross-modal Stroop effect (Xiao et al., 2014). In addition, behavioral evidence suggests that unimodal and cross-modal Stroop effects manifest themselves differently: the cross-modal Stroop effect appears smaller than the unimodal Stroop effect (Elliott et al., 2014); and the unimodal and cross-modal Stroop effects display a different pattern of development and decline (Hirst et al., 2019). That being said, more research would be required to reconcile these different results.

A P150-250 component was found in all conditions of the current study (i.e., NI, NC, PI, and PC conditions). This component is similar to a classic P2: both have the same latency (between 150 and 250 ms post-stimulus) and a similar scalp distribution (Carretié et al., 2001). The classic P2 is considered as a critical sign of attentional response to emotional visual stimuli. In our study, participants were required to categorize smells of the plants in the images. In order to respond accurately, participants had no choice but to focus on the images of the plants that varied on each basis. Therefore, it is possible that this ERP component is related to the same general visual information processing mechanism as the classic P2.

Interestingly, the NC condition elicited a larger P150-250 component than NI condition; and the PI condition elicited a larger P150-250 component than the PC condition. Participants saw images of pungent plants in the NC and PI conditions, whereas they saw images of aromatic plants in the PC and NI conditions. Thus, a greater P150-250 was evoked when participants saw images of pungent plants compared to images of aromatic plants. Previous studies found that the amplitude of the P2 increased as the attentional resources required to process the visual input increased (Correll et al., 2002; Ito and Bartholow, 2009). This led them to propose that the P2 is modulated by attention. Relatedly, studies on the effect of stimulus valence on ERP components also reported larger P2 amplitudes for negative relative to neutral stimuli (Carretié et al., 2001; Huang and Luo, 2006). One possible explanation for our results is thus that the brain spends more attentional resources in processing images of plants associated to an unpleasant odor compared to plants associated with a pleasant odor. That being said, the images did not differ in terms of evoked emotions: all participants rated both types of images as neutral. Therefore, the effect on the P2 was not driven by differences in terms of evoked emotions but possibly by differences associated to the visual representation of these images such as their represented valence or some low-level attributes. More work would need to be done using more carefully controlled visual stimuli to clarify this effect.

Using event-related brain potentials in combination with an olfactory-visual cross-modal Stroop task, the present study found ERP components associated with the olfactory-visual Stroop effect and the modulatory impact of olfactory-induced emotional states on the olfactory-visual Stroop effect. An incongruent minus congruent negative difference component was observed between 350 and 550 ms after stimulus onset. This ND350-550 component was interpreted as reflecting the selective attention in the olfactory-visual Stroop effect. Interestingly, this component was observed only in the negative olfactory context. This suggests that the positive sensory-induced emotional state had a facilitatory effect on selective attention and cognitive control, which translated into a reduction of brain potentials associated with the cross-modal Stroop effect. In addition, the present study found a larger component (P150-250) in NC condition than in NI condition; and a larger component (P150-250) in PI condition than in PC condition. We postulated that this could reflect an increase of attentional resources being allocated to targets with negative olfactory attributes. It should be pointed out that the ERP results showed selective attention improvements related to positive olfactory-induced emotional states but that the ANOVA on response times failed to demonstrate any facilitatory effect at the behavioral level. It is possible that the behavioral measurements were less sensitive to the process of selective attention than the neural ones. Similar contradictions were discovered in prior experiments (Luo et al., 2011; Yuan et al., 2011).

Although ERP data allow for precise statements on the time course of the olfactory-visual Stroop effect, it lacks the spatial resolution required to investigate spatial cortical activation patterns. Further experiments should thus be conducted using fMRI to explore which brain areas are associated with the olfactory-visual cross-modal Stroop effect.

## Data Availability Statement

The datasets generated for this study are available on request to the corresponding author.

## Ethics Statement

The studies involving human participants were reviewed and approved by the Ethics Committee of Chongqing Medical University. The patients/participants provided their written informed consent to participate in this study.

## Author Contributions

XX designed this study. XX, MX, and CG performed the study. XX and MX analyzed the data and drafted the manuscript. XX, JJ, and ND-R reviewed and revised the manuscript.

## Conflict of Interest

The authors declare that the research was conducted in the absence of any commercial or financial relationships that could be construed as a potential conflict of interest.
